# Calibration procedure and biomechanical validation of an universal six degree-of-freedom robotic system for hip joint testing

**DOI:** 10.1186/s13018-023-03601-2

**Published:** 2023-03-03

**Authors:** Michal Rychlik, Georg Wendland, Michal Jackowski, Roland Rennert, Klaus-Dieter Schaser, Joerg Nowotny

**Affiliations:** 1 University Center of Orthopaedics, Trauma and Plastic Surgery, University Hospital Carl Gustav Carus, TU Dresden, Fetscherstraße 74, 01307 Dresden, Germany; 2 Centre for Translational Bone, Joint and Soft Tissue Research, Dresden, Germany; 3 IMA Materialforschung Und Anwendungstechnik GmbH, Dresden, Germany; 4 Institute of Applied Mechanics, Poznan University of Technology, Poznan, Poland

**Keywords:** Hip joint, Six degree-of-freedom robot, Biomechanics, Testing, Kinematics

## Abstract

**Purpose:**

Among various test methods for different human joints, the use of robot systems has attracted major interest and inherits the potential to become a gold standard in biomechanical testing in the future. A key issue associated with those robot-based platforms is the accurate definition of parameters, e.g., tool center point (TCP), length of tool or anatomical trajectories of movements. These must be precisely correlated to the physiological parameters of the examined joint and its corresponding bones. Exemplified for the human hip joint, we are creating an accurate calibration procedure for a universal testing platform by using a six degree-of-freedom (6 DOF) robot and optical tracking system for recognition of anatomical movements of the bone samples.

**Methods:**

A six degree-of-freedom robot (TX 200, Stäubli) has been installed and configured. The physiological range of motion of the hip joint composed of a femur and a hemipelvis was recorded with an optical 3D movement and deformation analysis system (ARAMIS, GOM GmbH). The recorded measurements were processed by automatic transformation procedure (created in Delphi software) and evaluated in 3D CAD system.

**Results:**

The physiological ranges of motion were reproduced for all degrees of freedom with the six degree-of-freedom robot in adequate accuracy. With the establishment of a special calibration procedure by using a combination of different coordinate systems, we were able to achieve a standard deviation of the TCP depending of the axis between 0.3 and 0.9 mm and for the length of tool between + 0.67 and − 0.40 mm (3D CAD processing) resp. + 0.72 mm to − 0.13 mm (Delphi transformation). The accuracy between the manual and robotic movement of the hip shows an average deviation between − 0.36 and + 3.44 mm for the points on the movement trajectories.

**Conclusion:**

A six degree-of-freedom robot is appropriate to reproduce the physiological range of motion of the hip joint. The described calibration procedure is universal and can be used for hip joint biomechanical tests allowing to apply clinically relevant forces and investigate testing stability of reconstructive osteosynthesis implant/endoprosthetic fixations, regardless of the length of the femur, size of the femoral head and acetabulum or whether the entire pelvis or only the hemipelvis will be used.

**Supplementary Information:**

The online version contains supplementary material available at 10.1186/s13018-023-03601-2.

## Introduction

Due to the upright posture and bipedal gait that have resulted during the human evolution process, the development of osteoarthritis in the hip joint can be considered as a causative consequence and is nowadays generally considered to be a one of the most frequent civilization diseases [1, 2]. In Germany, nearly 18% of adults aged 18 years and older report to suffer from osteoarthritis in the last 12 months, with the prevalence being higher in women (21.8%) than in men (13.9%). The percentage of osteoarthritis patients increases significantly with age, among those aged 65 and older, almost half of the female population (48.1%) and a third of men (31.2%) are affected [3]. In order to develop new treatment strategies, tailored to the individual underlying pathology, a profound understanding of the biomechanics is essential.

In the literature, there are various approaches studying biomechanics and kinematics of the hip. Bergmann et al. as well as other authors have impressively shown that the dynamic loads carried by the human hip joint exceed the weight of the body almost by four times during walking and may even reach eightfold increased values during jumping or stumbling [4, 5]. Some authors reproduce physiological movements and force loads in the joint [6–8], while others focus on researching different implant materials, for example, to determine the corrosion and emergence of metal particles released by friction of implant parts [9, 10]. However, there is still no consistent methodology in the literature for biomechanical testing of joints, especially those associated to the hip joint.

The first biomechanical simulators were developed in the 50’s of the last century [11]. Until their appearance, various types of test machines (usually standard strength testing machines) were utilized using standard test methods [12–16]. For the improvement of the reproducibility and documentation of the precision, some authors reported the use of different 3D measuring devices allowing the reconstruction of simple movements in the hip joint [17–20]. Others used more complex measurement systems equipped with additional elements to emulate or measure muscle response [21, 22]. The common challenge of these solutions is the limited ability to reproduce complex joint movements, the restricted possibility of active control and the difficulty of reconfiguration of the device for other types of joints, movements or loading forces [17, 20, 23–25].

As van Arkel et al. described in their study [16], many research laboratories have developed new methods to study hip joint biomechanics based on various techniques, such as digital image correlation [26, 27], real-time contact-pressure measurement [28, 29], optical tracking motion analysis [30, 31], 3D digital reconstructions combining CT scans and motion tracking [32, 33], custom built rigs in servo-hydraulic actuators/materials testing devices [16, 26, 34, 35] or 6 DOF robots with actuators [36, 37]. Interesting advanced systems were also developed by Colbrunn et al. [36] and Al-Haifi et al. [38]. They constructed a three-dimensional testing system based on a six degrees of freedom parallel robot platform (Steward platform). Colbrunn et al. added a rotary stage to their system as a seventh degree of freedom [36]. The use of six degrees of freedom robots in biomechanical studies has a relatively short history [39, 40]. Due to their kinematic abilities, computer control and high positioning repeatability, they are very useful for precisely reproducing anatomical movements in joints [39, 41–43] and therefore are becoming the new gold standard [39, 44]. There are already studies that have been answered clinical relevant questions by using a 6 DOF robots in the knee joint [45, 46].

While most of these biomechanical testing devices focused on limited range of motion under constant cyclic loads [26, 34, 47], only few of them allowed variable loads while simultaneously reproducing more complex physiological movements [16, 18]. This establishes a strong demand for a uniform and standardized test setup (universal joint kinemator), in order to study complex biomechanical testing approaches for different motion and kinematics of the hip joint. For precise control of the robot platforms, a clear definition of a coordinate system in relation to the robot and the joint is mandatory. This is a precondition for exact 3D analysis and measurement as well as transmission of variable forces [42].

The purpose of this study was therefore to establish a standardized calibration procedure for a hip joint testing platform, which is also transferable to other joints (s.c. ‘universal joint kinemator’). For this purpose, we have used quantitatively assessed parameters, i.e., range of motion (trajectory of the motion), joint rotation center (defined for robots as tool center point) and length of bone specimen. Precise determination of these parameters is an essential precondition for development and setup of a 6 DOF robots, potentially allowing to perform subsequent biomechanical experiments under reproducible, dynamic real-time conditions with physiologically measured loadings.

## Methods

### Six degree-of-freedom robotic system for testing hip joint

**Fig. 1:**
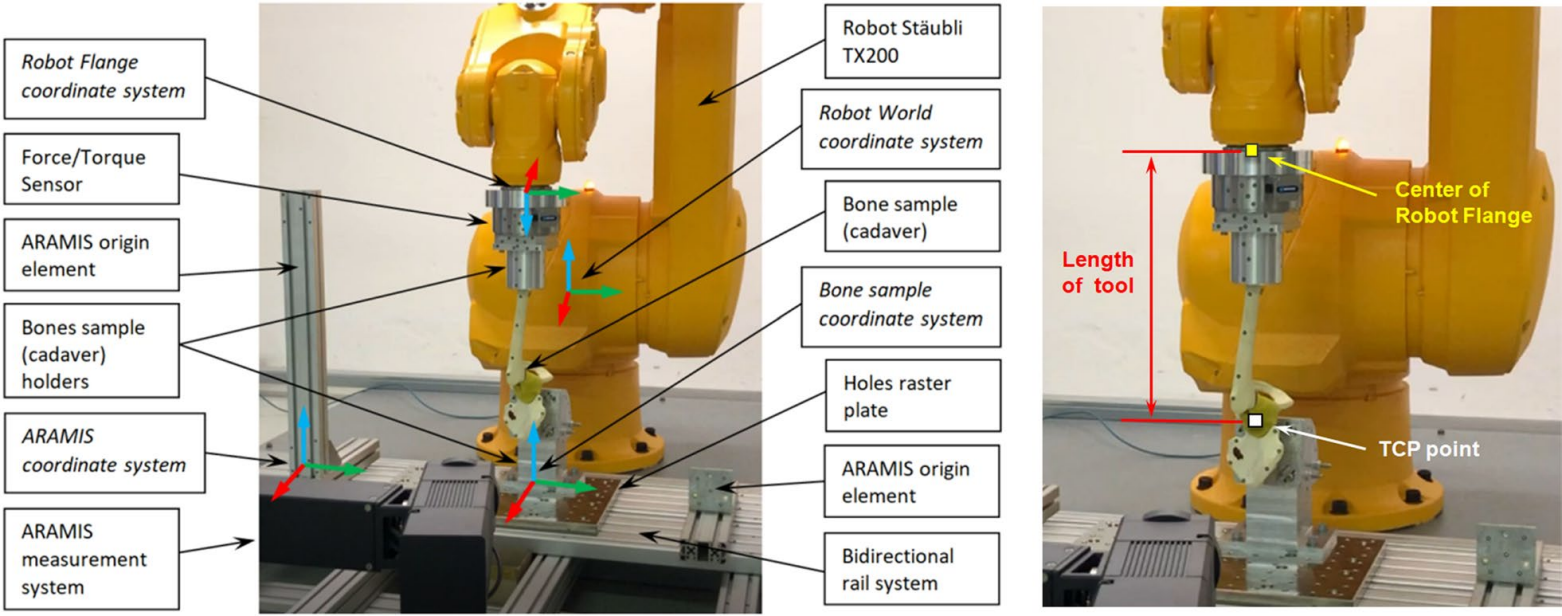
General view of the testing stand and the main elements with coordinate systems (left), presentation of the ‘length of tool’ (LOT) in relation to the ‘center of robot flange’ and the ‘tool center point’ (TCP) (right)

For the establishment of an ‘universal joint kinemator,’ a six degree-of-freedom robotic system (TX 200, Stäubli, Switzerland) was installed (Fig. 1), as it has been used for investigations of the knee and hip [41, 42]. With this robot, a maximum dynamic load of 1500 N and maximum static load of 9000 N can be applied, with a maximum range of 219.4 cm and a position repeatability (ISO 9283) of ± 0.06 mm [48]. In front of the robot, bidirectional (2 DOF—movements possible in *X* and *Y* direction) rail fastenings and a steel mounting plate with grid holes were installed to allow maximum variability of the object attachment. A force torque (FT) sensor (FTN-Omega160-IP-R15-NETB SI-1000–120, Schunk GmbH & Co. KG) with ± 1000 N maximum force measurement range in *X* and *Y* direction, ± 2500 N in *Z* direction and torque ± 120 Nm in R*x*, R*y* and R*z* direction (measurement resolution of forces: 0.25 N, and torques: 0.025 Nmm for R*x*, R*y* and 0.015 Nmm for R*z* direction) was additionally mounted on the robot flange to measure loads (Fig. 1).

### Samples

Artificial hemipelvis-femur-bone samples with normal anatomical proportions (right hip, foam cortical shell, SKU: 1172, medium size, Sawbones, Malmö, Sweden) were used for the testing of the robot movement. The distal femurs were removed 5 cm from the joint line with an oscillating saw to ensure a secure fixation in the holding adapter and were placed into bone cement (Dental Plaster Typ 4, Excalibur, water/plaster ratio 22:100, Siladent, Dr. Böhme & Schöps GmbH) in an exact position according to the mechanical axis of the femur in relation to the center of rotation of the hip joint. In terms of alignment, we have been oriented to the International Society of Biomechanics (ISB) recommendations for definition of joint coordinate systems [49]. The hemipelvis-femur was then installed on the robot testing setup. The distal femur (cemented in holder) was attached to the force torque sensor and the hemipelvis to the rail fastening system (Fig. 1). Subsequently, the fixation of the joint was removed, so that the joint could be moved manually for calibration of future movements by the robot.

A hemipelvis with native hip joint and complete femur from a male (81 years) specimen was used for the final investigation of the accuracy between manual and robotic movements (positive evaluation for the use of human specimens for biomechanical testing: EK 51,012,019).

### Photo-optical and kinematic calibration

Photo-optical measurements were carried out by a high-resolution 3D-stereo photo-optical system (ARAMIS ASX, GOM GmbH, Braunschweig, Germany) with a frequency up to 10 Hz, a measurement volume of 1100 × 800 × 500 mm and an accuracy of 0.001 mm to evaluate the axes and finally the precision of the movements. The system was aligned and positioned at 90° to the sagittal plane (*y*-axis) of the hemipelvis-femur according to the recommended orientation conditions [49].

### Establishment of the coordinate systems

The spatial understanding of these coordinate systems is essential, since a conversion of the coordinates between each other is necessary for the transformation of the motion between the investigated specimen and the robot. The structure of the ‘universal joint kinemator’ has four main coordinate systems defined in the Cartesian space related to the basic elements (Fig. 1):

- Robot world coordinate system—associated with the robot base,
- Robot flange coordinate system—associated with the robot flange (sixth axis),
- ARAMIS coordinate system—associated with the optical ARAMIS measuring system,
- Bone sample coordinate system—associated with the testing bone sample.

The Robot World coordinate system of the TX 200 Stäubli is located on the first moveable element (rotation between the robot base and the robot shoulder) of the robot (Fig. 1) [48]. Additionally connected with the Robot World coordinate system is the so-called tool center point (TCP) specifying the tool tip position (point around which the robot arm moves) of the robot tool and corresponds to the joint rotation center (JRC). ‘Tool Length’ is related to the distance between the TCP point (JRC) and center of robot flange (Fig. 1), which corresponds to the most distal movement point of the robot. Practice shows that most often the TCP point does not coincide with the *Z*-axis of robot flange but is spatially shifted in relation to it. The ARAMIS coordinate system is associated with a set of three groups of optical markers (two on the left and one on the right ARAMIS origin element) that form the reference system (Fig. 1). The bone sample coordinate system is related to the bone sample mounted to the bidirectional rail system (Fig. 1).

### Determination of motion parameters of manual movements (stage I)

**Fig. 2:**
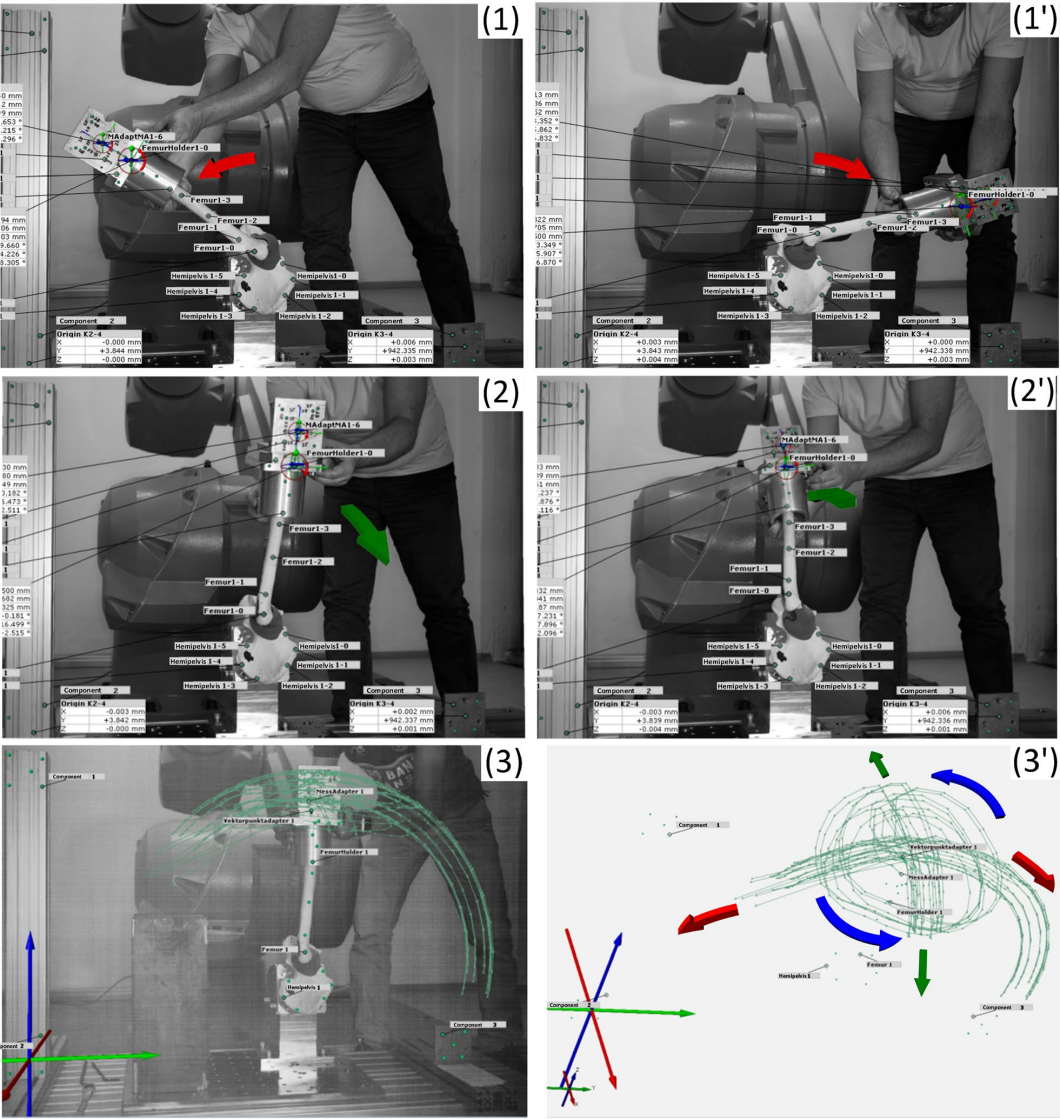
Calibration Stage I—Evaluation of the manual movements of hemipelvis femur bone sample (Sawbone) with mounted calibration adapter: flexion–extension (**1**), (**1ꞌ**), abduction–adduction (**2**), (**2ꞌ**), visualization of femur trajectory for all movements including circular movements (**3**), (**3ꞌ**) images recorded by ARAMIS system

The aim of stage I is to determinate motion parameters of the specimen plane (bone sample coordinate system) by using standardized hemipelvis-femur-bone samples (Sawbones, Malmö, Sweden). For this purpose, the trajectories of physiological anatomical movements (range of motion—ROM) were evaluated and used to define the TCP. The femur was moved manually without use of external forces (preloads) and the range of motion was recorded with the ARAMIS system (Fig. 2). During the test, four types of physiological movements were measured: flexion–extension, abduction–adduction, external and circular movement of femur. The collected data was obtained from the measurement system in data sets of *X*-, *Y*-, *Z* coordinates and angles of rotations *α*, *β* and *γ*. To obtain correct data, a specially configured ‘calibration adapter’ (aluminum element) with 3D/6 DOF definition and precisely marked points (seven markers of the ARAMIS system) was manufactured (Fig. 4, bottom) and mounted on the top of the femur holder (Fig. 2).

**Fig. 3:**
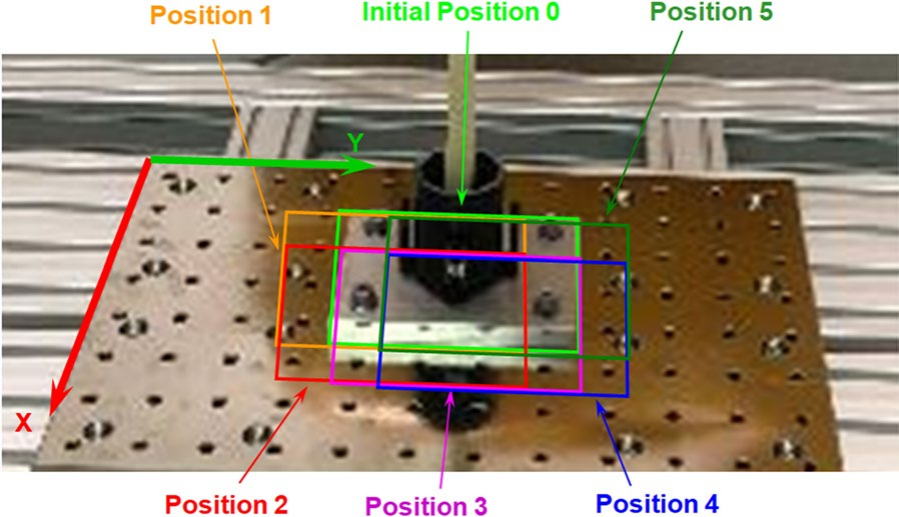
The varying settings of the robot test platform during the experiment with six different mounting positions of the bone sample (base plate with holes raster 50 × 50 mm)

To evaluate the accuracy of the setup for the bone sample coordinate system, the tests were repeated in six different positions with an offset of 50 mm from the initial position, while maintaining the full range of motion of the robot (Fig. 3). This results in a change in the position and tilt angle of the Aramis system, while maintaining the correct measurement range of the system in relation to the robot and the examined bone sample.

### Determination of motion parameters of the robot (stage II)

**Fig. 4:**
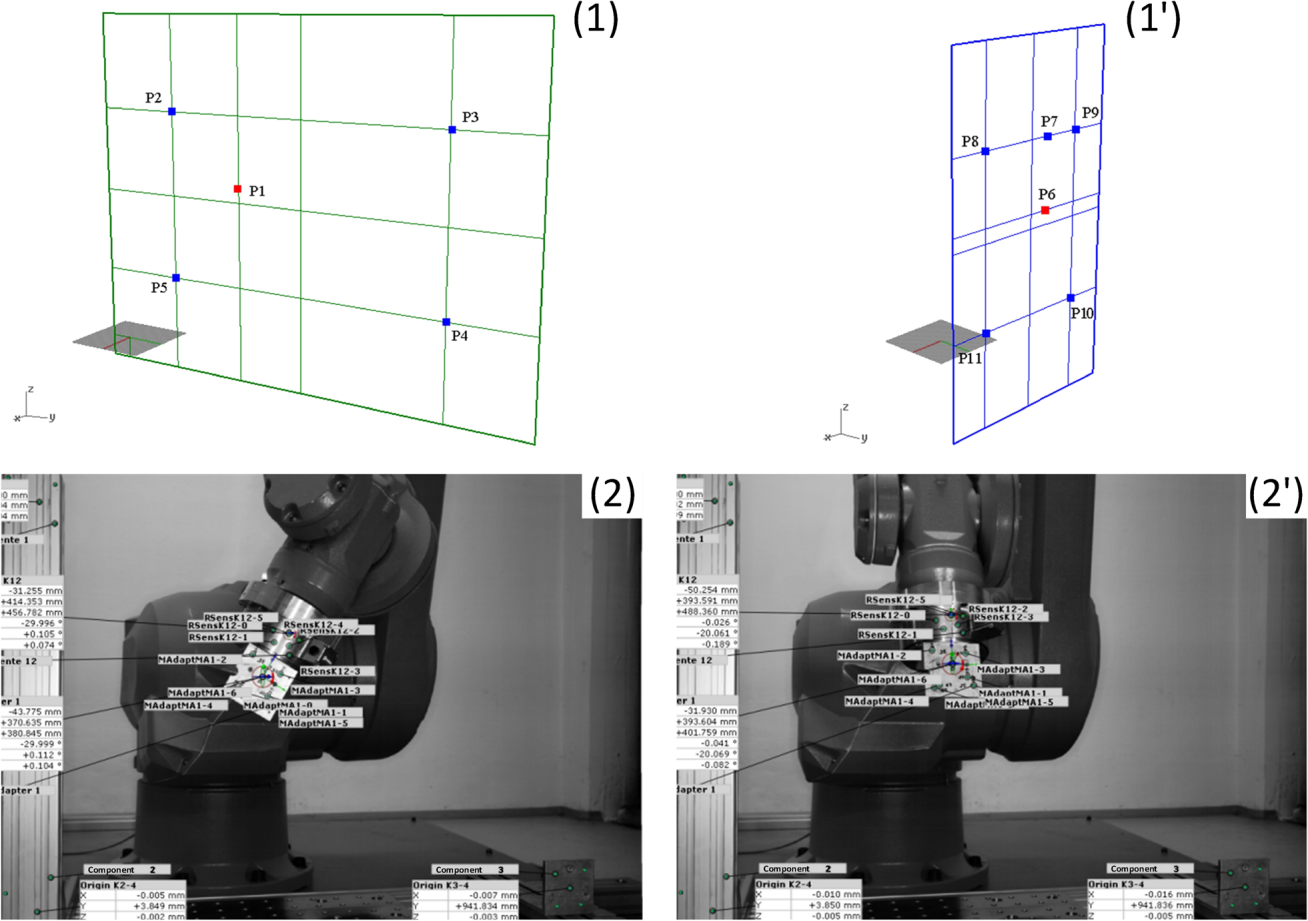
Stage II calibration of the robot: calibration points P1–P5 localized on plane YZ (**1**), calibration points P6–P11 localized on plane XZ (**1ꞌ**), examples of robot flange manipulations—two different positions (**2**) and (**2ꞌ**), with mounted calibration adapter (images recorded by ARAMIS system)

For second stage calibration process, the ‘Calibration adapter’ (see stage I) was mounted to the robot flange (Fig. 4, (2)). As a primary step, the robot's YZ plane was defined and measured, by moving the robot to five different localizations in space (points: P1–P5) documented optically by ARAMIS system (Fig. 4, (1)). As a variable different rotations of the robot flange around, these points were used, while all points were localized on constant *X* value (rotation performed sequentially at individual points P1-P11 in a sequence of rotations of the robot flange around the *X*-, *Y*- and *Z*-axis).

In a secondary step, the *XZ* plane was defined as a vertical axis localized at the intersection of the *YZ* axes and through the point P1 (from step 1). The procedure was repeated analogously to the previous one generating the points P6–P11 (Fig. 4, (1’)).

### Evaluation of tool center point and length of tool (stage III)

The main objective of stage III was to obtain fundamental parameters for the definition of the position of the tool center point (TCP) and the length of tool (LOT) in the robot world coordinate system and therefore consequently in the coordinate system of the robot control panel. For evaluation of the calibration process, the following parameters were determined:

- Distance between calibration points (P1–P5 in YZ and P6–P11 in XZ robot plane) in comparison with the theoretical set of coordinates in the robot control unit, defining the error of measuring and reconstructing the position of the robot flange during calibration procedure
- Reconstructed Robot World origin (point *x*R, *y*R, *z*R = 0, 0, 0),
- Maximum deviation of the center of the Robot World origin,
- Main localization of the Robot World origin (defined as an average value of all calibrations points) related to the ARAMIS origin.

### Establishment of a numerical transformation for robot controlling (Delphi transformation)

The data obtained from the measurements using the ARAMIS optical system were automatically processed (by geometric transformations) in an automated transformation procedure developed for this purpose (in the Delphi computer programming language). The automatic transformation procedure allows the transformation of the input data from the calibration to obtain the following data: conversion of ARAMIS coordinates into Robot world coordinates, length of tool (length of bone sample, LOT), movements trajectory of the bone sample around the joint, TCP defined in Robot world coordinates and the error of the obtained results (Figs. 8 and 10).

**Fig. 5:**
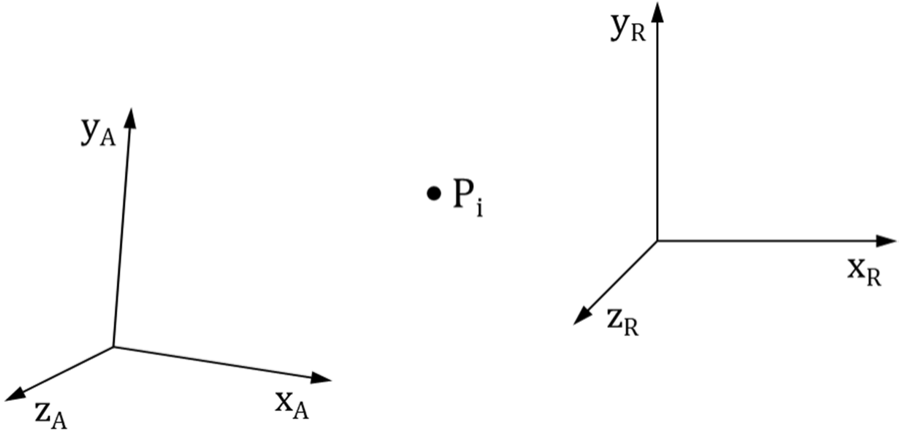
Bases of ARAMIS—and robot world coordinate system

For the analysis of the calibration measurement, digital measurement data are given for the different robot positions *i* = 1,…,*n*, in robot world coordinates $x_{R,i} ;y_{R,i} ;z_{R,i}$ as well as in ARAMIS coordinates $x_{A,i} ;y_{A,i} ;z_{A,i}$ (Fig. 5).

We aimed to determine a transformation matrix to convert the coordinates in any robot position from one coordinate system into the other one. This transformation was carried out in two steps, separated into translation shift and rotation shift of both coordinate systems. The translation shift of both coordinate systems is performed quite simply by calculation of the coordinate differences of both measured coordinate sets with the first data set *i* = 1 of the calibration measurement, where all coordinates are shifted to a Zero-point. With the robot control values of the first date set of the calibration measurement $x_{R,1} ;y_{R,1} ;z_{R,1}$, it follows the zeroed robot coordinates and the zeroed ARAMIS coordinates.

$$\begin{array}{*{20}c} {\tilde{x}_{R,i} = x_{R,i} - x_{R,1} } & {\tilde{x}_{A,i} = x_{A,i} - x_{A,1} } \\ \end{array}$$

$$\begin{array}{*{20}c} {\tilde{y}_{R,i} = y_{R,i} - y_{R,1} } & {\tilde{y}_{A,i} = y_{A,i} - y_{A,1} } \\ \end{array}$$

$$\begin{array}{*{20}c} {\tilde{z}_{R,i} = z_{R,i} - z_{R,1} } & {\tilde{z}_{A,i} = z_{A,i} - z_{A,1} } \\ \end{array}$$

The rotation matrix is calculated via matrix operations. For that purpose, the available measurement data are collected in two 3 × n-matrices, where n is the total number of the calibration measurement data sets.

Both zeroed measurement data matrices are

$$\begin{array}{*{20}c} {\underline {R} = \left[ {\begin{array}{*{20}c} {\tilde{x}_{R,1} } & {\tilde{x}_{R,2} } & \cdots & {\tilde{x}_{R,n} } \\ {\tilde{y}_{R,1} } & {\tilde{y}_{R,2} } & \cdots & {\tilde{y}_{R,n} } \\ {\tilde{z}_{R,1} } & {\tilde{z}_{R,2} } & \cdots & {\tilde{z}_{R,n} } \\ \end{array} } \right]} & {\underline {A} = \left[ {\begin{array}{*{20}c} {\tilde{x}_{A,1} } & {\tilde{x}_{A,2} } & \cdots & {\tilde{x}_{A,n} } \\ {\tilde{y}_{A,1} } & {\tilde{y}_{A,2} } & \cdots & {\tilde{y}_{A,n} } \\ {\tilde{z}_{A,1} } & {\tilde{z}_{A,2} } & \cdots & {\tilde{z}_{A,n} } \\ \end{array} } \right]} \\ \end{array}$$

The relation between both measurement data sets is defined by the—initially unknown—transformation matrix $\underline {T}_{A \to R}$, where index *A* → *R* indicates the transformation direction from ARAMIS coordinates into robot coordinates:

$$\underline {R} = { }\underline {T}_{A \to R} \cdot \underline {A}$$

For solution of this equation to the transformation matrix, at first the transposed matrix $\underline {A}^{T}$ needs to be multiplied from the right side:

$$\underline {R} \cdot \underline {A}^{T} = { }\underline {T}_{A \to R} \cdot \underline {A} \cdot \underline {A}^{T}$$

Because the resulting 3 × 3-matrix $\underline {A} \cdot \underline {A}^{T}$ is general invertible, it can be continued with

$$\underline {R} \cdot \underline {A}^{T} \cdot \left( {\underline {A} \cdot \underline {A}^{T} } \right)^{ - 1} = { }\underline {T}_{A \to R} \cdot \underline {A} \cdot \underline {A}^{T} \cdot \left( {\underline {A} \cdot \underline {A}^{T} } \right)^{ - 1}$$

Afterward because of $\underline {A} \cdot \underline {A}^{T} \cdot \left( {\underline {A} \cdot \underline {A}^{T} } \right)^{ - 1} = \underline {E}$, the equation for the calculation of the transformation matrix from ARAMIS coordinates into robot coordinates follows as

$$\underline {T}_{A \to R} = \underline {R} \cdot \underline {A}^{T} \cdot \left( {\underline {A} \cdot \underline {A}^{T} } \right)^{ - 1}$$

The transformation matrix in the opposite direction from robot coordinates into ARAMIS coordinates is the inverse matrix

$$\underline {T}_{R \to A} = \left( {\underline {T}_{A \to R} } \right)^{ - 1}$$

### Determination of sphere equation of the tested hip joint

**Fig. 6:**
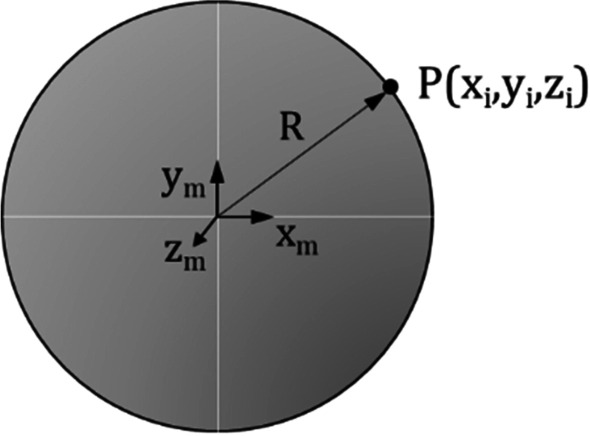
Sphere parameters *x*_*m*, *y*_*m*, *z*_*m*, *R* and one hip joint measurement data set ${x}_{i},{y}_{i},{z}_{i}$

The hip joint measurements delivered measurement data sets $x_{i} ;y_{i} ;z_{i}$ of possible positions of the center point of the robot adapter plate. If the trajectory of the measured data is similar to a spheroidal shape, only the parameters of the related sphere—middle point $x_{m} ;y_{m} ;z_{m}$ and radius $R$ of the sphere (Fig. 6) need to be given to the control system of the robot.

If the trajectory of the measured data is not similar to a spheroidal shape, only the trajectory of an ‘allowed coordinate trace’ can be given to the control system of the robot. The proof of the likelihood of the hip joint measurement data to a spheroidal shape is carried out by the following procedure, which can be performed using ARAMIS coordinates as well as in robot coordinates since the conversion between both coordinate system is possible with the procedure as described above. In general, a sphere can be described by equation

$$\left( {x - x_{m} } \right)^{2} + \left( {y - y_{m} } \right)^{2} + \left( {z - z_{m} } \right)^{2} = R^{2}$$

which can be expanded to

$$x^{2} - 2 \cdot x \cdot x_{m} + x_{m}^{2} + y^{2} - 2 \cdot y \cdot y_{m} + y_{m}^{2} + z^{2} - 2 \cdot z \cdot z_{m} + z_{m}^{2} = R^{2}$$.

The sorting of all terms with the parameters of the sphere $x_{m}$ $y_{m}$ $z_{m}$ and R to the left side of the equation and all other terms to the right side of the equation leads to

$$x_{m}^{2} + y_{m}^{2} + z_{m}^{2} - R^{2} - 2 \cdot x_{m} \cdot x - 2 \cdot y_{m} \cdot y - 2 \cdot z_{m} \cdot z = - \left( {x^{2} + y^{2} + z^{2} } \right)$$.

The factors related to 1, *x, y,* and *z* on the left side of the equation can be substituted to

$$p_{1} = x_{m}^{2} + y_{m}^{2} + z_{m}^{2} - R^{2}$$

$$p_{2} = - 2 \cdot x_{m}$$

$$p_{3} = - 2 \cdot y_{m}$$

$$p_{4} = - 2 \cdot z_{m}$$.

With these substitutions, it follows

$$p_{1} + p_{2} \cdot x + p_{3} \cdot y + p_{4} \cdot z = - \left( {x^{2} + y^{2} + z^{2} } \right)$$

The four unknown factors $p_{1}$ $p_{2}$ $p_{3}$ and $p_{4}$ require at least four equations. These four equations can be obtained by a sufficient repetition number of the hip joint measurement. If measurement data sets of more than four hip joint measurements are available, these measurements can be written as matrices

$$\left[ {\begin{array}{*{20}c} 1 & {x_{1} } & {y_{1} } & {z_{1} } \\ 1 & {x_{2} } & {y_{2} } & {z_{2} } \\ \vdots & \vdots & \vdots & \vdots \\ 1 & {x_{i} } & {y_{i} } & {z_{i} } \\ \vdots & \vdots & \vdots & \vdots \\ 1 & {x_{n} } & {y_{n} } & {z_{n} } \\ \end{array} } \right] \cdot \left[ {\begin{array}{*{20}c} {p_{1} } \\ {p_{2} } \\ {p_{3} } \\ {p_{4} } \\ \end{array} } \right] = \left[ {\begin{array}{*{20}c} { - \left( {x_{1}^{2} + y_{1}^{2} + z_{1}^{2} } \right)} \\ { - \left( {x_{2}^{2} + y_{2}^{2} + z_{2}^{2} } \right)} \\ \vdots \\ { - \left( {x_{i}^{2} + y_{i}^{2} + z_{i}^{2} } \right)} \\ \vdots \\ { - \left( {x_{n}^{2} + y_{n}^{2} + z_{n}^{2} } \right)} \\ \end{array} } \right]$$

, respectively, $$\underline {K} \cdot \underline {p} = \underline {R}$$.

The searched parameter vector $\underline {p}^{T} = \left[ {p_{1} ,p_{2} ,p_{3} ,p_{4} } \right]$ can be calculated by use of the pseudo-inverse matrix via.

$$\underline {p} = \left( {\underline {K}^{T} \underline{ \cdot K} } \right)^{ - 1} \cdot \underline {K}^{T} \cdot \underline {R} .$$

The parameters of the sphere equation $x_{m}$, $y_{m} ,$ $z_{m}$ and *R* result from the backward substitutions

$$x_{m} = - p_{2} /2$$

$$y_{m} = - p_{3} /2$$

$$z_{m} = - p_{4} /2$$

$$R = \sqrt {x_{m}^{2} + y_{m}^{2} + z_{m}^{2} - p_{1} } .$$

While this matrix operation is very fast and effective, the analysis of the measurement data sets from the hip joint measurements did not lead to useful results. A possible reason might be a partially very unfavorable condition number of the linear equation system as well as the disturbance by some inaccurate data sets of the hip joint measurement.

To overcome this problem, the calculation of the sphere equation was separated into all possible four-point combinations of the entire hip joint measurement. That means, if the entire hip joint measurement consists of $n$ single measurements, all these single measurements will be combined into all their possible four-point combinations. For example, if only five single measurements exist, the possible four-point combination is:

1. Measurement 1, measurement 2, measurement 3, measurement 4
2. Measurement 1, measurement 2, measurement 3, measurement 5
3. Measurement 1, measurement 2, measurement 4, measurement 5
4. Measurement 1, measurement 3, measurement 4, measurement 5

In general, if $n$ single measurements exist, the total number of possible four-point-combinations is factorial of $n$ divided by factorial of four:

$$n!/4! = n!/24 .$$

For each combination, the four equations for the calculation of the sphere equation were solved

$$p_{1} + p_{2} \cdot x_{1} + p_{3} \cdot y_{1} + p_{4} \cdot z_{1} = - \left( {x_{1}^{2} + y_{1}^{2} + z_{1}^{2} } \right)$$

$$p_{1} + p_{2} \cdot x_{2} + p_{3} \cdot y_{2} + p_{4} \cdot z_{2} = - \left( {x_{2}^{2} + y_{2}^{2} + z_{2}^{2} } \right)$$

$$p_{1} + p_{2} \cdot x_{3} + p_{3} \cdot y_{3} + p_{4} \cdot z_{3} = - \left( {x_{3}^{2} + y_{3}^{2} + z_{3}^{2} } \right)$$

$$p_{1} + p_{2} \cdot x_{4} + p_{3} \cdot y_{4} + p_{4} \cdot z_{4} = - \left( {x_{4}^{2} + y_{4}^{2} + z_{4}^{2} } \right)$$.

**Fig. 7:**
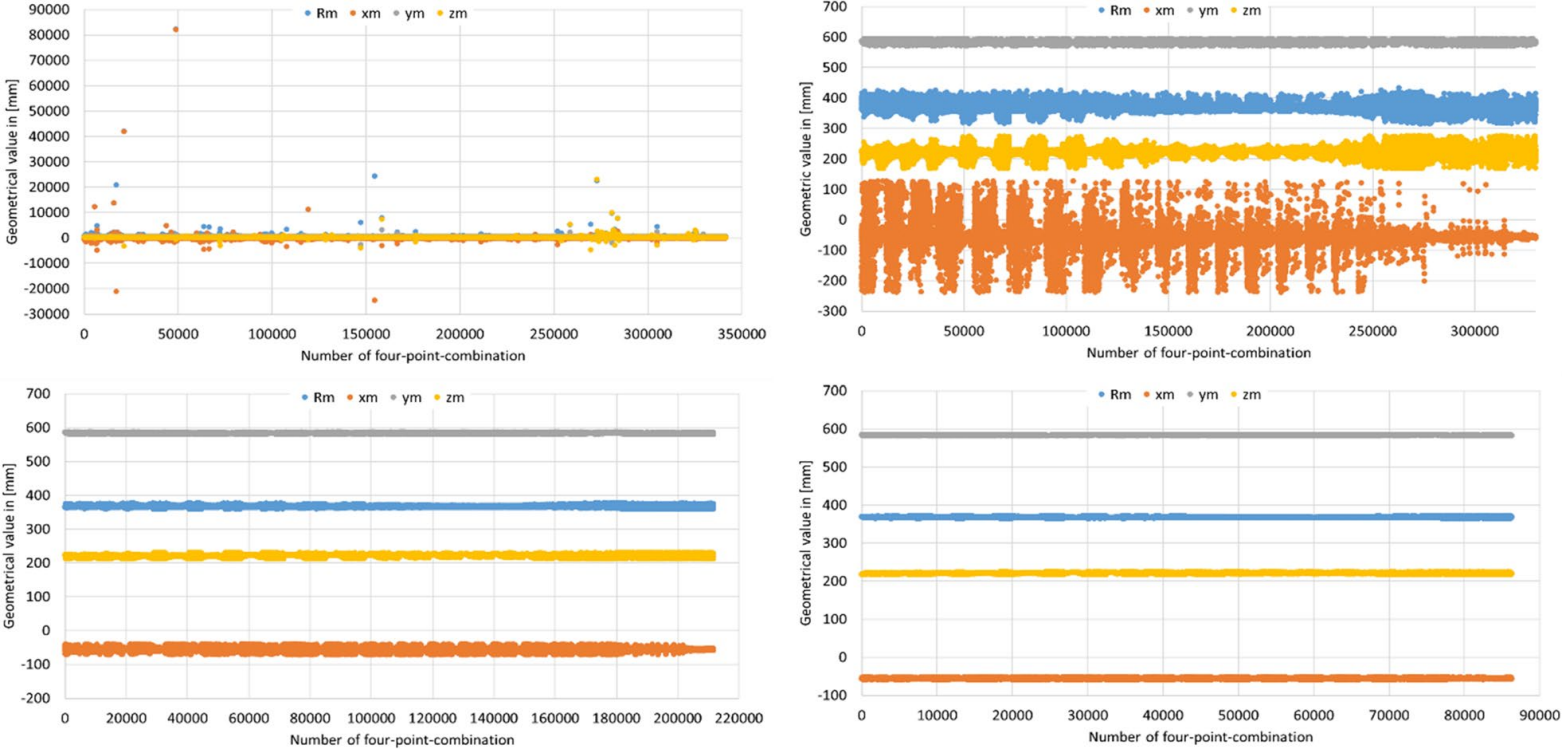
Parameters of the sphere equation from four-point combination

Substitution and backward substitution of $p_{1}$, $p_{2}$, $p_{3}$ and $p_{4}$ was carried out as described before. In result, the parameters of the sphere equation have been calculated for each four-point combination of the hip joint measurement (Fig. 7, upper left). As can be seen, some single four-point combinations led to huge errors in the calculation of the sphere equation parameters. To exclude them from the further analysis, the mean value and the standard deviation of all parameters were calculated. All results were removed, which exceeded a defined threshold of plus/minus standard deviation from the mean value. The result of the first application of this threshold rule is depicted in Fig. 7 (upper right). While the result of the first application of the threshold rule was still not sufficient, the same rule was applied subsequently for a third (Fig. 7, bottom left) and fourth time, see Fig. 7 (bottom right).

After this repeated application of the threshold rule, analysis results close to the median of all remaining results. These results were used to calculate the arithmetic average of equation $x_{m}$, $y_{m}$, $z_{m}$ and $R$ which are considered as the resulting parameters of the sphere equation.

Furthermore, the standard deviation of all remaining single values of $x_{m}$, $y_{m}$, $z_{m}$ and $R$ was calculated. If these standard deviations are small, the trajectory of the hip joint measurement is similar to a sphere shape. If these standard deviations are large, the trajectory of the hip joint measurement is not similar to a sphere shape. The decision, which standard deviation can be evaluated as ‘small’ or ‘large,’ depends on the specified tolerances for the following hip joint test.

In addition, 3D CAD illustration (Rhinoceros 3D, Robert McNeel & Associates) was used to visualize the input data and, respectively, the evaluated results. Subsequently, the evaluated data of the bone sample calibration procedures were processed, to identify the TCP of the bone sample (artificial hemipelvis-femur).

### Evaluation of accuracy between manual (calibration) and robotic (automatic reproduction) movement:

**Fig. 8:**
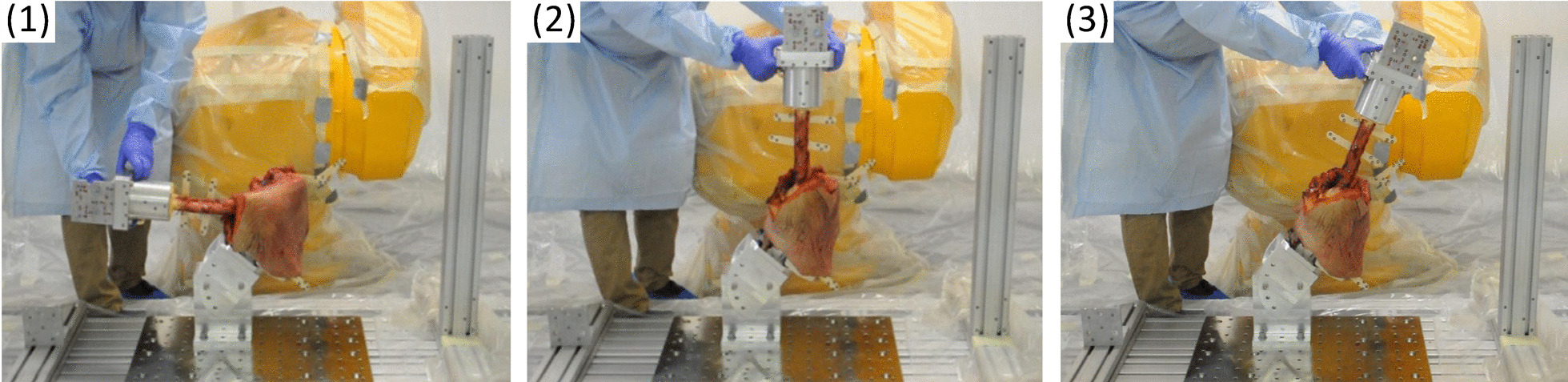
Documentation of manual flexion–extension of the human hip joint: images (**1**), (**2**), (**3**) present three selected positions from the movement

Based on the proposed methodology, a motion reproduction for human hip joint specimens was performed. Therefore, a human hemipelvis cadaver was mounted on the test platform by using special holders. Primarily, the trajectories of the physiological movements of the hip were evaluated (stage I). The femur was moved manually (without external force) and the physiological range of motion was recorded with the ARAMIS system (Fig. 8). The motion was recorded with a frequency of 10 Hz, which allows for a large number of measurement points with good reproduction of the pathway.

Secondary, the robot's calibration movements were performed according to the aforementioned procedure. The robot's movements are always the same and are determined in the robot's control program according to calibration points P1–P11 (stage II).

In the next step, the developed numerical transformation (Delphi Transformation) was used to convert the coordinates of the manual movement of the human hip specimen (recorded in optical ARAMIS coordinates system), into the Robot world coordinate system, which is necessary to control the robot's movement adequately (stage III).

**Fig. 9:**
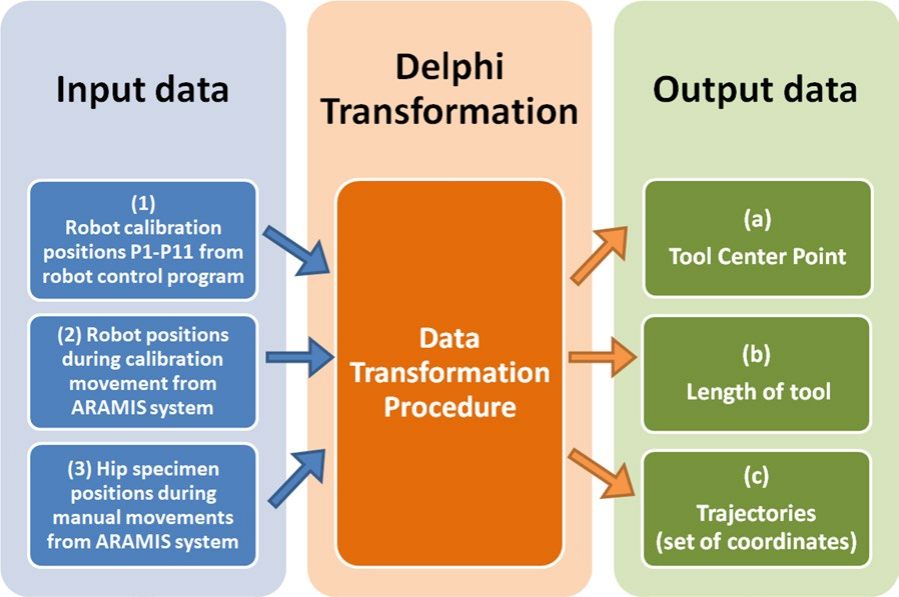
Block diagram of the Delphi Transformation: (1) input data of robot calibration positions P1-P11 from robot control program, (2) input data of robot positions during calibration movement from ARAMIS system, (3) input data of hip specimen manual movements from ARAMIS system, (a) output data of TCP, (b) output data of length of tool (LOT), (c) output data of coordinates points (trajectories) transformed into robot world coordinate system

Three data sets were used as input for the Delphi transformation (Fig. 9):

1. Coordinates from the robot control program including positions of the robot for the calibration procedure—points P1–P11 (data in robot world coordination system)
2. Coordinates of the calibration plate attached to the robot flange measured during robot calibration movements done by the ARAMIS optical measurement system (points recorded in the ARAMIS coordinate system)
3. Coordinates of the markers of the calibration plate attached to the femur bone sample holder obtained during manual movement (points recorded in the ARAMIS coordinate system).

As the results of Delphi Transformation, the following output is obtained (Fig. 9, Additional file 1):

- TCP which corresponds to the JRC,
- LOT which corresponds to the length of the femur bone with the holder,
- Trajectories (set of coordinates) of the femur cadaver movement transformed into robot world coordinate system.

The reconstruction of the anatomical movements can be achieved using data from the Delphi Transformation in two ways:

Method 1 uses a designated TCP and LOT. This method is reserved solemnly for joints that can be defined as ball joints (i.e., the hip joint). In the presented investigation, the coordinates of the TCP were determined as *x*R = 1009.45, *y*R = − 124.54, *z*R = 59.39 (coordinates written in the Robot world coordinate system), while the LOT was evaluated to be 383.11 mm.

Method 2 uses data in the form of a trajectory of motion of the end of the femur bone (farthest tracker from the JRC), without using the JRC and without using definition of tool length (TCP set as value 0 mm + length of force torque sensor). This method is more universal and can be applied to various types of joints, e.g., the elbow joint. In the described investigation, a series of coordinates (*x*R, *y*R, *z*R) were obtained for more than 230 points showing the movement of flexion–extension, abduction–adduction and circular movement of the femur.

**Fig. 10:**
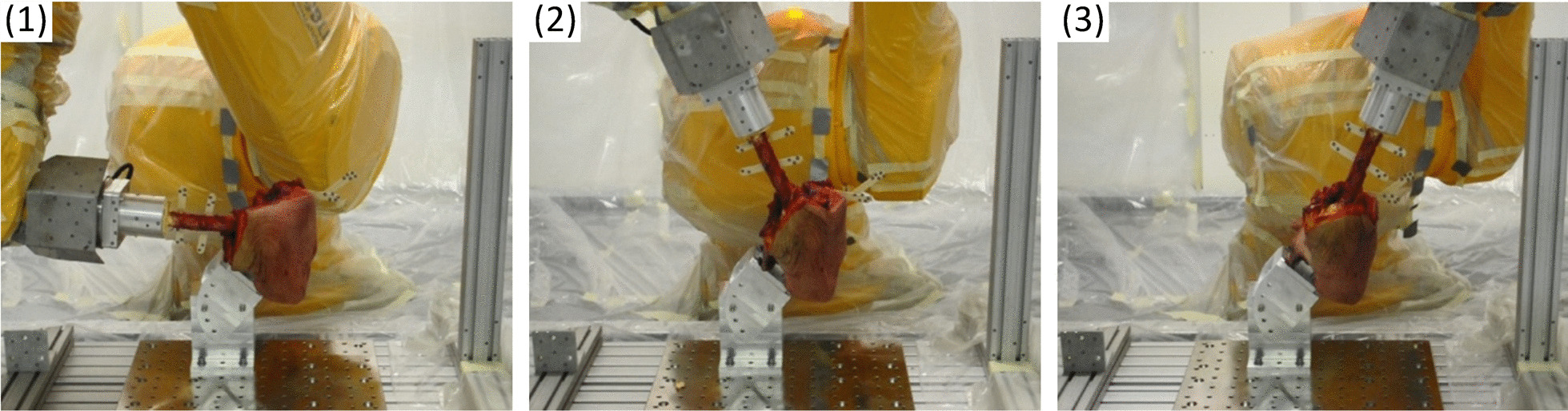
Physiological movements of the human hip specimen reproduced by the robot for flexion–extension: images (**1**), (**2**), (**3**) present three selected positions from the movement

The output data obtained from the Delphi Transformation (either according to method 1 or 2) is entered into the robot control system. The robot is then positioned by the operator at a point coinciding with a single point in the motion trajectory. Next, the attachment of the femur holder to the robot flange is performed. Now the physiological movements in automatic mode are feasible (Fig. 10).

To evaluate the quality of the robot's movement reproduction, measurements of the robot's automatic movements were made and analyzed using the same optical ARAMIS system. All measurement procedures with the ARAMIS system were performed on the same day of the investigation, with equal conditions and in the same position according to the robot. For better understanding and visualization of the obtained results, the trajectories of movements during manual calibration and robotic motion were compiled and analyzed in a three-dimensional CAD system.

## Results

### Determination of the accuracy (repeatability) of designation of the reference point of the world robot coordinate system

In the present study, reconstruction of Robot World origin was created individually for each of the six measurement positions (Fig. 3). For this purpose, the *x*A, *y*A, *z*A reference point coordinates measured by the ARAMIS optical system for all six bone sample mounting positions were compared with each other. The average value of deviation around the theoretical position of Robot world origin *x*R, *y*R, *z*R = 0, 0, 0 was  ± 0.3 mm. The maximum values did not exceed ± 0.7 mm (Table 1).

**Table 1:** Average values of deviation for reconstruction of the Robot World origin—values based on all six measurement positions

Direction	*x*R	*y*R	*z*R
Position 0	− 0.003	0.378	0.218
Position 1	− 0.002	0.667	0.126
Position 2	0.000	0.104	− 0.025
Position 3	0.001	− 0.159	0.041
Position 4	0.184	− 0.241	0.178
Position 5	0.001	− 0.089	0.005
Average value	0.030	0.110	0.091
Standard deviation	0.069	0.321	0.126
Max deviation	0.184	0.667	0.218
Min deviation	− 0.003	− 0.241	− 0.025

### Accuracy of calibration procedure of robot parameters TCP and LOT

#### Tool center point—TCP (hip rotation center)

Since the fixed holder of the bone samples was shifted by six various positions on the mounting plate (Fig. 3), the values obtained for the TCP also reflect these different spatial positions. For easier interpretation of the results, the data were normalized according to the shift values resulting from the 50 × 50 mm hole matrix and the fixing positions of the bone sample holder (Table 2).

**Table 2:** TCP position normalized to Position 0,0,0 in World Robot coordination system for six positions of bone samples (all values in mm)

Direction	*x*R	*y*R	*z*R
Position 0	1064.00	97.02	− 201.19
Position 1	1059.64	96.45	− 200.66
Position 2	1058.72	95.69	− 199.79
Position 3	1061.02	95.15	− 201.05
Position 4	1061.25	95.07	− 200.22
Position 5	1059.90	97.93	− 199.14
Standard deviation	1.68	1.03	0.72
Average value	1060.76	96.22	− 200.34
Max value '+'	1064.00	97.93	− 199.14
Min value '−'	1058.72	95.07	− 201.19

In addition, the data with the TCP coordinates were compiled and transformed with the values of the main setting in the robot control program. The deviations were adjusted to a mean value of deviation at zero. The obtained results represent the deviation values of TCP position as defined in the robot control program (Table 3).

**Table 3:** Deviation of the TCP position referred to the settings in robot control program (all values in mm)

Direction	*x*R	*y*R	*z*R
Position 0	− 0.393	− 0.016	− 0.100
Position 1	− 1.618	0.651	0.037
Position 2	0.380	− 0.181	0.486
Position 3	0.159	− 0.497	− 0.465
Position 4	0.360	− 0.951	0.148
Position 5	1.110	0.995	− 0.108
Standard deviation	0.847	0.658	0.288
Max deviation '+'	1.110	0.995	0.486
Min deviation '−'	− 1.618	− 0.951	− 0.465

#### Length of tool (LOT)

The last parameter analyzed was the determination of the tool length (Table 4) which defines the distance between the TCP and the center of robot flange after attachment of the femur holder to the robot (Fig. 1).

**Table 4:** Results of the tool length (LOT) determination for bone sample (all values in mm)

Direction	*x*R	*y*R	*z*R	3 D CAD	Delphi transformation
Position 0	25.737	− 0.005	365.337	366.242	367.96
Position 1	26.137	− 1.822	365.124	366.063	367.69
Position 2	25.777	− 0.326	365.467	366.375	366.54
Position 3	25.353	− 1.013	365.626	366.505	367.37
Position 4	25.380	− 0.270	365.600	366.480	366.79
Position 5	24.015	0.963	366.347	367.135	367.10
Average value	25.400	− 0.412	365.584	366.467	367.24
Median deviation	0.159	0.114	− 0.050	− 0.039	0.48
Max deviation '+'	0.737	1.375	0.763	0.668	0.72
Min deviation '−'	1.385	1.410	0.459	0.404	0.13

The average value for the LOT measured with 3D CAD was approximately 366.47 mm (including the length of the femur bone sample together with the femur holder) with median the range of deviation not exceeding 0.04 mm. The extreme values of deviations are about maximum/minimum deviation of + 0.67 mm and − 0.4 mm, respectively. Similarly, values could also be determined for the Delphi transformation, where the LOT was approx. 367.24 mm with mean deviation of 0.48 and the maximum/minimum deviation of 0.72 mm and 0.13 mm.

### Comparative investigation of the accuracy between manual and robotic movement with hip specimens

**Fig. 11:**
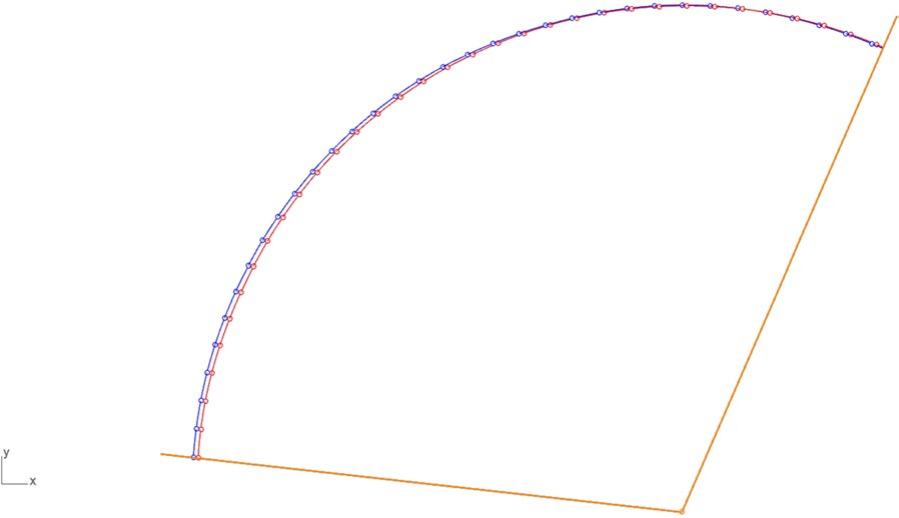
Comparison of motion trajectories recorded for flexion–extension movement: manual motion of the bone sample recorded during calibration (red curve), reconstruction of the motion of the bone sample made by the robot (blue curve)

In the final stage of the investigation, the quality of the motion trajectory was compared between manual and robotic movement (Figs. 8, 10 and 11, Table 5). For each movement, the deviations of the manual trajectory curve points *x*A, *y*A, *z*A (recorded by Aramis during calibration) vs. the curve points reproduced by the robot in the *x*R, *y*R, *z*R axis directions were evaluated. In each case, the average, maximum and minimum deviation of the points of the compared curves were determined.

**Table 5:** Results of the trajectory curve coordinates deviation recorded for flexion–extension motion

	d*X*	d*Y*	d*Z*	Euclidean distance
Average	3.44	− 0.36	0.17	3.59
Max	3.66	0.24	1.50	3.79
Min	3.26	− 0.59	− 1.57	3.41

The results obtained lead to the conclusion that the highest deviation of the movement trajectory can be evaluated for the *x*-axis. The average deviation of the points on the trajectory curves varies between 3.44 and 0.17 mm, with the lowest deviation in the *z*-axis and the highest in *x*-axis direction. The maximum deviation with 3.66 mm was also in the *x*-axis, while the lowest (0.24 mm) was evaluated in the *y*-axis. The average value for the Euclidean distance in 3D space was obtained at 3.59 mm (value determined for all *X*, *Y*, *Z* coordinates).

## Discussion

Biomechanical and kinematic understanding of complex joints (i.e., the hip joint) is essential for further comprehension of pathologies, injuries and surgical procedures. The main limitation of common biomechanical investigations is the mostly uniplanar force or tensile application [50–53]. Therefore, 3D motion and force input is necessary for measuring complex movement patterns and kinematics [4, 5, 54]. The use of a 6 DOF robots guarantees the possibility of free motion in 3D-space while maintaining high positioning repeatability [41, 44, 55, 56]. Additionally, the opportunity of developing appropriate control algorithms allows reproducibility of the complex 3D movements of the test sample in accordance with the anatomical structure of the examined joint and bone. This will lead to a better understanding of the biomechanics and kinematics of the hip joint and, in the long term, may help to investigate endoprostheses biomechanically and make them safer for patients.

The main focus of the current study was to develop an accurate and fast calibration method for this testing approach by using a robotic platform without the need for detailed measurements of the geometrical joint parameters (femoral head, acetabulum) and characteristic points of specimens (to determine the axes defined by the ISB). Therefore, an algorithm was developed to establish a universal applicable biomechanical platform using a numerical transformation. Within the presented calibration, measurements are obtained using an optical measurement system and data processing takes about 10 min to generate the coordinates (trajectory) for robot movements.

We were able to show that by combining different coordinate systems, a standard deviation of the TCP between 0.3 and 0.9 mm depending on the axis could be achieved. The maximum deviation of the LOT was evaluated between + 0.67 and − 0.4 mm (3D CAD processing) and 0.72–0.13 mm (Delphi transformation). The accuracy between the manual and robotic movement of the hip shows an average deviation between − 0.36 and + 3.44 mm for the points on the movement trajectories. The presented error represents the values as a total error for the entire ‘universal kinemator’ system. The total error results from adding up error of control and positioning of the robot, measurement error of the optical measurement (ARAMIS) system, the error in defining individual planes and axes of the robot and bone samples, the error of joint imperfections and error in defining the calibration element and its mounting.

Goldsmith et al. examined the reproducibility by using the hip joint model and was able to demonstrate that a 6 DOF robotic system allows to identify hip passive paths (without external loads) in a highly repeatable manner (median RMS error of < 0.1 mm and < 0.4 degrees) [41, 42]. Moreover, the robotically simulated clinical exams were found to be consistent and repeatable (rotational RMS error ≤ 0.8 degrees) in determining hip ranges of motion. After converting the degree values to our specified LOT, the deviation seems to be similar in the underlying study (1 degree of error results in a position error of about 3.5 mm at the end of the femur). However, the indication of the maximum deviation appears to be superior, since maximum divergent robot trajectory can cause possible peak forces with potential damage to the specimen under investigation. Compared to Goldsmith et al. in the aforementioned method, there is no need to measure the femoral head or hip acetabulum for defining the position of the JRC relative to the bone landmarks [41]. In addition, there is no need for intraarticular preparation and no risk of damaging the hip joint and subsequently altering its biomechanical parameters, which may have negative effects on TCP when a best-fit sphere is used to describe the locations recorded on the surface of the femoral head with the intact capsule [41].

In order to ensure correct fixation and load on the examined hip joint, it is necessary to properly define the individual anatomical and mechanical axes of the tested sample, as well as define the hip joint center [49]. For determination of HJC, special procedures and protocols must be used [57]. The calibration procedure presented in the current study allows to define basic parameters such as HJC (robot definition: TCP), geometric dimensions of the bone sample mounted in the handles (robot definition: LOT) without risk of accidental damage to the joint/bone sample or other elements of the test set up. The advantage of our proposed method in comparison with others is that the movement can be mimicked by two methods, using the TCP, the length of tool (based on an approximation of the shape of the joint to a sphere) or using the recorded physiological movement trajectory of the femur [41, 55]. Moreover, in most setups a compressive force (axially and medially 10 N) is required to ensure contact between the femoral head and the articular surface of the acetabulum [42]. In our methodology, this is not necessary (measurements are taken without external forces) since the entire joint is intact and provides full anatomical mobility.

While there are no consistent calibration procedures in terms of the establishment of biomechanical platforms using a 6 DOF, the current study demonstrates a transparent and universal applicable calibration pathway for further research approaches. The proposed innovative trajectory-based approach is a universal solution independent of the shape of the joint. As a result, the method can be used to accurately represent joint function, regardless of the fact that the femoral head is naturally conchoidal and slightly aspherical in shape [58]. This can be particularly important when performing tests on pathological joints (i.e., after trauma). This approach also has the advantage of making the system robust to inaccuracies in the mounting of the specimens in the grips relative to the planes and the axes defined using the ISB coordinate system [49].

A limitation of our study is the small number of bone samples examined and thus associated failure to account for the variables of age and gender. The recorded inaccuracies in the realization of the movements may be due to some systematic error in the whole system, including errors in movement registration, robot calibration, sample mounting, data processing and generation of the control program for the robot. In addition, the effect of changing the length of the test sample (LOT) to the error of trajectory reconstruction was not defined. In the underlying study, the length of the femur including the handle was close to 367 mm, which may also affect the deviation values. These values can be different in comparison with tests presented by other researches where the length of tool is much shorter [55]. However, with the current method, it is possible to perform complex movements in different axes simultaneously—for example, rotational movement around the JRC.

## Conclusion

A six degree-of-freedom robot is appropriate to reproduce the physiological range of motion in hip joint. The described procedure of calibration is universal and can be used for hip joint biomechanical tests, regardless of the length of the femur, size of the femoral head and acetabulum or whether the entire pelvis or hemipelvis is used.

Clinical implications of this universal calibration procedure may include further robot studies that are aimed to apply physiological loading forces [4, 54]. The use and translation of these in vivo measured data, indicating force transmission and loads in the joints during activities of daily living, allows much more realistic biomechanical stability testing of endoprosthetic implants and osteosynthesis [54]. Pursuing this approach may add much more value to the currently used and commonly available test procedures. These and other potential applications of the procedure for other kinematic and biomechanical joint examinations will be the subject of further investigations.

## Supplementary Information

**Additional file 1.** Delphi Transformation (screenshot).

## Data Availability

The material and the data are made available.

## Abbreviations

3 D: Three dimensional
DOF: Degree of freedom
e.g.: Latin: ‘exempli gratia’; English: for example
Fig.: Figure
FT: Force torque
HJC: Hip joint center
ISB: International Society of Biomechanics
LOT: Length of tool
max.: Maximum
min.: Minimum
mm: Millimeter
N: Newton
SD: Standard deviation
TCP: Tool center point
vs.: Versus

## References

[1] Biegański P, Polewska E. Choroba zwyrodnieniowa stawów biodrowych—pacjent i problemy funkcjonalne = The hip joint Osteoarthritis—patient and functional problems. 2015 [Cited 2022 Sep 4]; Available from: https://zenodo.org/record/22712

[2] Ziemlanska B, Nowicki T, Market K. Comparison of clinical and radiological symptoms in patients with hip osteoarthritis qualified for arthroplasty. Ann Acad Med Gedanensis. 2011;41:17–25.

[3] Fuchs J, Kuhnert R, Scheidt-Nave C. 12-Monats-Prävalenz von Arthrose in Deutschland. J Health Monit. Robert Koch-Institut Epidemiol und Gesundh. 2017. 10.17886/RKI-GBE-2017-054

[4] Bergmann G, Bender A, Dymke J, Duda G, Damm P (2016). Standardized loads acting in hip implants. PLoS ONE.

[5] Bergmann G, Graichen F, Rohlmann A, Bender A, Heinlein B, Duda GN (2010). Realistic loads for testing hip implants. Biomed Mater Eng.

[6] Fraysse F, Dumas R, Cheze L, Wang X (2009). Comparison of global and joint-to-joint methods for estimating the hip joint load and the muscle forces during walking. J Biomech.

[7] Maletsky LP, Hillberry BM (2005). Simulating dynamic activities using a five-axis knee simulator. J Biomech Eng.

[8] Stansfield BW, Nicol AC, Paul JP, Kelly IG, Graichen F, Bergmann G (2003). Direct comparison of calculated hip joint contact forces with those measured using instrumented implants. An evaluation of a three-dimensional mathematical model of the lower limb. J Biomech.

[9] Crackau M, Märtens N, Harnisch K, Berth A, Döring J, Lohmann CH (2020). In vivo corrosion and damages in modular shoulder prostheses. J Biomed Mater Res B Appl Biomater.

[10] Lee J, Salvati E, Betts F, DiCarlo E, Doty S, Bullough P (1992). Size of metallic and polyethylene debris particles in failed cemented total hip replacements. J Bone Jt Surg Br.

[11] Hicks JH (1953). The mechanics of the foot. I. The joints. J Anat.

[12] Anderson AE, Ellis BJ, Maas SA, Peters CL, Weiss JA. Validation of finite element predictions of cartilage contact pressure in the human hip joint. J Biomech Eng. 2008;130(5):051008. 10.1115/1.2953472 10.1115/1.2953472 PMC2840996 19045515

[13] Debski RE, McMahon PJ, Thompson WO, Woo SL-Y, Warner JJP, Fu FH (1995). A new dynamic testing apparatus to study glenohumeral joint motion. J Biomech.

[14] Hewitt J, Guilak F, Glisson R, Vail TP (2001). Regional material properties of the human hip joint capsule ligaments. J Orthop Res.

[15] Satpathy J, Kannan A, Owen JR, Wayne JS, Hull JR, Jiranek WA (2015). Hip contact stress and femoral neck retroversion: a biomechanical study to evaluate implication of femoroacetabular impingement. J Hip Preserv Surg.

[16] van Arkel RJ, Amis AA, Jeffers JRT (2015). The envelope of passive motion allowed by the capsular ligaments of the hip. J Biomech.

[17] Bay BK, Hamel AJ, Olson SA, Sharkey NA (1997). Statically equivalent load and support conditions produce different hip joint contact pressures and periacetabular strains. J Biomech.

[18] Burroughs BR, Hallstrom B, Golladay GJ, Hoeffel D, Harris WH (2005). Range of motion and stability in total hip arthroplasty with 28-, 32-, 38-, and 44-mm femoral head sizes. J Arthroplast.

[19] Chandler DR, Glousman R, Hull D, McGuire PJ, Kim IS, Clarke IC, et al. Prosthetic hip range of motion and impingement. The effects of head and neck geometry. Clin Orthop Relat Res. 1982;(166):284–91. 7083681

[20] Ferguson SJ, Bryant JT, Ganz R, Ito K (2003). An in vitro investigation of the acetabular labral seal in hip joint mechanics. J Biomech.

[21] Delp SL, Hess WE, Hungerford DS, Jones LC (1999). Variation of rotation moment arms with hip flexion. J Biomech.

[22] Heinrichs CH, Knierzinger D, Stofferin H, Schmoelz W (2018). Validation of a novel biomechanical test bench for the knee joint with six degrees of freedom. Biomed Eng Biomed Tech.

[23] Crawford MJ, Dy CJ, Alexander JW, Thompson M, Schroder SJ, Vega CE (2007). The 2007 frank Stinchfield award: the biomechanics of the hip labrum and the stability of the hip. Clin Orthop Relat Res.

[24] Smith MV, Costic RS, Allaire R, Schilling PL, Sekiya JK (2014). A biomechanical analysis of the soft tissue and osseous constraints of the hip joint. Knee Surg Sports Traumatol Arthrosc.

[25] Walker PS, Blunn GW, Broome DR, Perry J, Watkins A, Sathasivam S (1997). A knee simulating machine for performance evaluation of total knee replacements. J Biomech.

[26] Dickinson AS, Taylor AC, Browne M (2012). The influence of acetabular cup material on pelvis cortex surface strains, measured using digital image correlation. J Biomech.

[27] Dickinson AS, Taylor AC, Ozturk H, Browne M. Experimental validation of a finite element model of the proximal femur using digital image correlation and a composite bone model. J Biomech Eng. 2011;133(1):014504. 10.1115/1.4003129 10.1115/1.4003129 21186906

[28] Lee S, Wuerz TH, Shewman E, McCormick FM, Salata MJ, Philippon MJ (2015). Labral reconstruction with iliotibial band autografts and semitendinosus allografts improves hip joint contact area and contact pressure. Am J Sports Med.

[29] Rudert MJ, Ellis BJ, Henak CR, Stroud NJ, Pederson DR, Weiss JA, Brown TD (2014). A new sensor for measurement of dynamic contact stress in the hip. J Biomech Eng.

[30] Lopomo N, Sun L, Zaffagnini S, Giordano G, Safran MR (2010). Evaluation of formal methods in hip joint center assessment: an in vitro analysis. Clin Biomech.

[31] Signorelli C, Lopomo N, Bonanzinga T, Marcheggiani Muccioli GM, Safran MR, Marcacci M (2013). Relationship between femoroacetabular contact areas and hip position in the normal joint: an in vitro evaluation. Knee Surg Sports Traumatol Arthrosc.

[32] Dwyer MK, Jones HL, Hogan MG, Field RE, McCarthy JC, Noble PC (2014). The acetabular labrum regulates fluid circulation of the hip joint during functional activities. Am J Sports Med.

[33] Incavo SJ, Thompson MT, Gold JE, Patel RV, Icenogle KD, Noble PC (2011). Which procedure better restores intact hip range of motion: total hip arthroplasty or resurfacing? A combined cadaveric and computer simulation study. J Arthroplasty.

[34] Song Y, Ito H, Kourtis L, Safran MR, Carter DR, Giori NJ (2012). Articular cartilage friction increases in hip joints after the removal of acetabular labrum. J Biomech.

[35] van Arkel RJ, Amis AA, Cobb JP, Jeffers JRT (2015). The capsular ligaments provide more hip rotational restraint than the acetabular labrum and the ligamentum teres. Bone Jt J.

[36] Colbrunn RW, Bottros JJ, Butler RS, Klika AK, Bonner TF, Greeson C (2013). Impingement and stability of total hip arthroplasty versus femoral head resurfacing using a cadaveric robotics model. J Orthop Res.

[37] Smith MV, Panchal HB, Ruberte Thiele RA, Sekiya JK (2011). Effect of acetabular labrum tears on hip stability and labral strain in a joint compression model. Am J Sports Med.

[38] Joyce TJ. The design and development of a finger joint simulator. Proc Inst Mech Eng H. 2016;230(5):450–7. 10.1177/0954411915626943 10.1177/0954411915626943 26833697

[39] Fujie H, Mabuchi K, Woo SL-Y, Livesay GA, Arai S, Tsamot Y (1993). The use of robotics technology to study human joint kinematics: a new methodology. J Biomech Eng.

[40] Li G, Rudy TW, Sakane M, Kanamori A, Ma CB, Woo SL-Y (1999). The importance of quadriceps and hamstring muscle loading on knee kinematics and in-situ forces in the ACL. J Biomech.

[41] Goldsmith MT, Rasmussen MT, Turnbull TL, Trindade CAC, LaPrade RF, Philippon MJ (2015). Validation of a six degree-of-freedom robotic system for hip in vitro biomechanical testing. J Biomech.

[42] Goldsmith MT, Smith SD, Jansson KS, LaPrade RF, Wijdicks CA (2014). Characterization of robotic system passive path repeatability during specimen removal and reinstallation for in vitro knee joint testing. Med Eng Phys.

[43] Rudy TW, Livesay GA, Woo SL-Y, Fu FH (1996). A combined robotic/universal force sensor approach to determine in situ forces of knee ligaments. J Biomech.

[44] Herrmann S, Kluess D, Kaehler M, Grawe R, Rachholz R, Souffrant R (2015). A novel approach for dynamic testing of total hip dislocation under physiological conditions. PLoS ONE.

[45] Ball S, Stephen JM, El-Daou H, Williams A, Amis AA (2020). The medial ligaments and the ACL restrain anteromedial laxity of the knee. Knee Surg Sports Traumatol Arthrosc.

[46] Kebbach M, Grawe R, Geier A, Winter E, Bergschmidt P, Kluess D (2019). Effect of surgical parameters on the biomechanical behaviour of bicondylar total knee endoprostheses: a robot-assisted test method based on a musculoskeletal model. Sci Rep.

[47] Sangiorgio SN, Longjohn DB, Lee JL, Alexander JD, Dorr LD, Ebramzadeh E (2008). Simulation of extreme loads on the proximal femur for implant fixation assessment. J Appl Biomater Biomech.

[48] Stäubli. Stäubli manual. Roboterbaureihe TX200: Industrieroboter–6 Achsen. 2019.

[49] Wu G, Siegler S, Allard P, Kirtley C, Leardini A, Rosenbaum D (2002). ISB recommendation on definitions of joint coordinate system of various joints for the reporting of human joint motion—part I: ankle, hip, and spine. J Biomech.

[50] Osterhoff G, Tiziani S, Hafner C, Ferguson SJ, Simmen H-P, Werner CML (2016). Symphyseal internal rod fixation versus standard plate fixation for open book pelvic ring injuries: a biomechanical study. Eur J Trauma Emerg Surg.

[51] Freitas A, Maciel RA, Lima RDA, Souto DRDM, Ferrer MDA (2014). Mechanical analysis of femoral neck fracture fixation with dynamic condylar screw in synthetic bone. Acta Ortop Bras.

[52] Philippon MJ, Rasmussen MT, Turnbull TL, Trindade CAC, Hamming MG, Ellman MB (2014). Structural properties of the native ligamentum teres. Orthop J Sports Med.

[53] Stoffel K, Zderic I, Gras F, Sommer C, Eberli U, Mueller D (2017). Biomechanical evaluation of the femoral neck system in unstable Pauwels III femoral neck fractures: a comparison with the dynamic hip screw and cannulated screws. J Orthop Trauma.

[54] Damm P, Kutzner I, Bergmann G, Rohlmann A, Schmidt H (2017). Comparison of in vivo measured loads in knee, hip and spinal implants during level walking. J Biomech.

[55] el Daou H, Ng KCG, van Arkel R, Jeffers JRT, Rodriguezy Baena F (2019). Robotic hip joint testing: development and experimental protocols. Med Eng Phys.

[56] Lenarcic MS (2010) Journal of Advances in robot kinematics Motion in man and machine. USA: Springer Dordrecht Heidelberg London New York.

[57] Camomilla V, Cereatti A, Vannozzi G, Cappozzo A (2006). An optimized protocol for hip joint centre determination using the functional method. J Biomech.

[58] Menschik F (1997). The hip joint as a conchoid shape. J Biomech.

