# Supplementary material for: Calibration procedure and biomechanical validation of an universal six degree-of-freedom robotic system for hip joint testing
Source: J Orthop Surg Res. 2023 Mar 3;18:164. doi: 10.1186/s13018-023-03601-2 (PMC9983254; doi:10.1186/s13018-023-03601-2)
Supplement: Supplementary file 1 — Additional file 1. Delphi Transformation (screenshot). [file 13018_2023_3601_MOESM1_ESM.docx]

**Additional material**


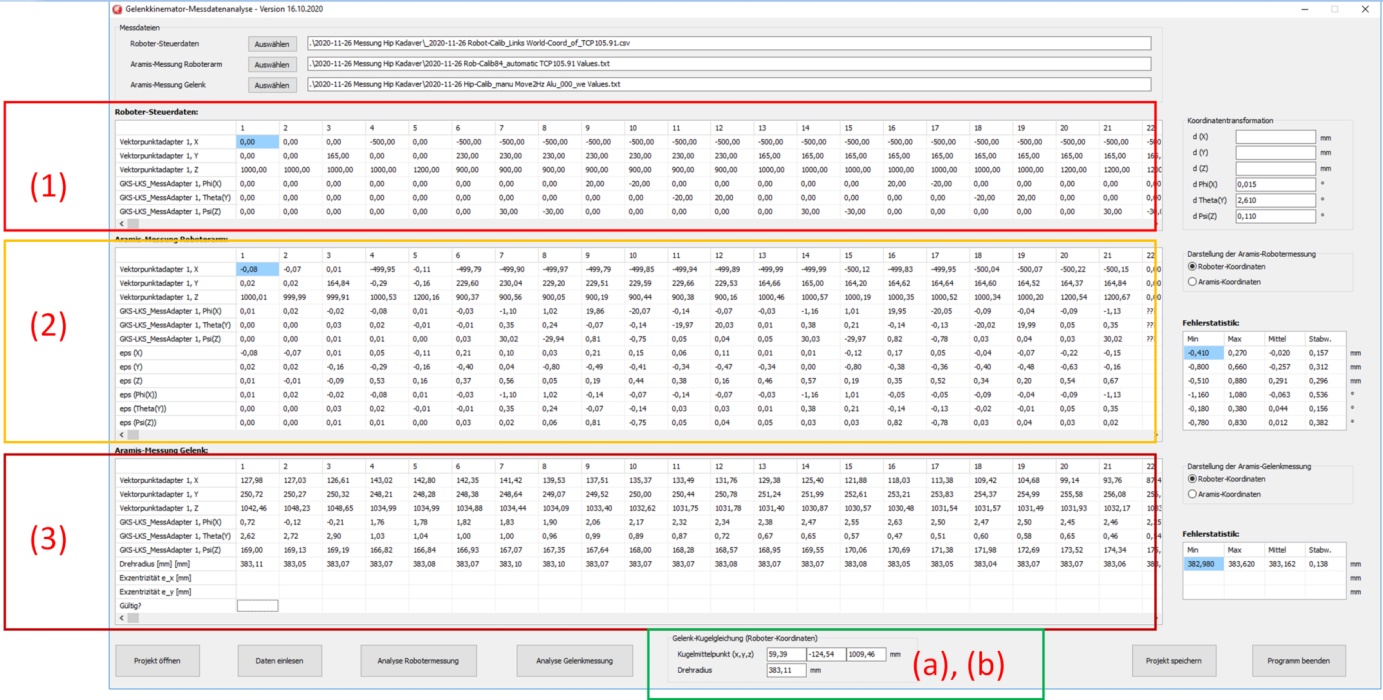


Figure 13: Delphi Transformation (screenshot): (1) input data of robot calibration positions P1-P11 from robot control program, (2) input data of robot positions during calibration movement from ARAMIS system, (3) input data of hip specimen manual movements from ARAMIS system,
(a) output data of TCP, (b) output data of length of tool (LOT)
